# Electronic structures of MgO/Fe interfaces with perpendicular magnetization revealed by hard X-ray photoemission with an applied magnetic field

**DOI:** 10.1080/14686996.2019.1633687

**Published:** 2019-06-18

**Authors:** Shigenori Ueda, Masaki Mizuguchi, Masahito Tsujikawa, Masafumi Shirai

**Affiliations:** a Research Center for Advanced Measurement and Characterization, National Institute for Materials Science, Tsukuba, Japan; b Synchrotron X-ray Station at SPring-8, National Institute for Materials Science, Hyogo, Japan; c Institute for Materials Research, Tohoku University, Sendai, Japan; d Center for Spintronics Research Network, Tohoku University, Sendai, Japan; e CREST, Japan Science and Technology Agency, Kawaguchi, Japan; f Research Institute of Electrical Communication, Tohoku University, Sendai, Japan; g Center for Science and Innovation in Spintronics (Core Research Cluster), Tohoku University, Sendai, Japan

**Keywords:** Electronic structures, hard X-ray photoelectron spectroscopy (HAXPES), HAXPES under a magnetic field, MgO/Fe interface, perpendicular magnetic anisotropy (PMA), interface-induced PMA, 40 Optical, magnetic and electronic device materials, 203 Magnetics / Spintronics / Superconductors, 212 Surface and interfaces, 502 Electron spectroscopy

## Abstract

We have developed hard X-ray photoelectron spectroscopy (HAXPES) under an applied magnetic field of 1 kOe to study the electronic and magnetic states related to the MgO/Fe interface-induced perpendicular magnetic anisotropy (PMA). In this work, we used MgO (2 nm)/Fe (1.5 and 20 nm)/MgO(001) structures to reveal the interface-induced electronic states of the Fe film. Perpendicular magnetization of the 1.5-nm-thick Fe film without extrinsic oxidation of the Fe film was detected by the Fe 2*p* core-level magnetic circular dichroism (MCD) in HAXPES under a magnetic field, and easy magnetization axis perpendicular to the film plane was confirmed by *ex situ* magnetic hysteresis measurements. The valence-band HAXPES spectrum of the 1.5-nm-thick Fe film revealed that the Fe 3*d* electronic states were strongly modified from the thick Fe film and a reference bulk Fe sample due to the lifting of degeneracy in the Fe 3*d* states near the MgO/Fe interface. We found that the tetragonal distortion of the Fe film by the MgO substrate also contributes to the lifting of degeneracy in the Fe 3*d* states and PMA, as well as the Fe 3*d*-O 2*p* hybridization at the MgO/Fe interface, by comparing the valence-band spectrum with density functional theory calculations for MgO/Fe multilayer structures. Thus, we can conclude that the Fe 3*d*-O 2*p* hybridization and tetragonal distortion of the Fe film play important roles in PMA at the MgO/Fe interface. HAXPES with *in situ* magnetization thus represents a powerful new method for studying spintronic structures.

## GRAPHICAL ABSTRACT

**Figure:**
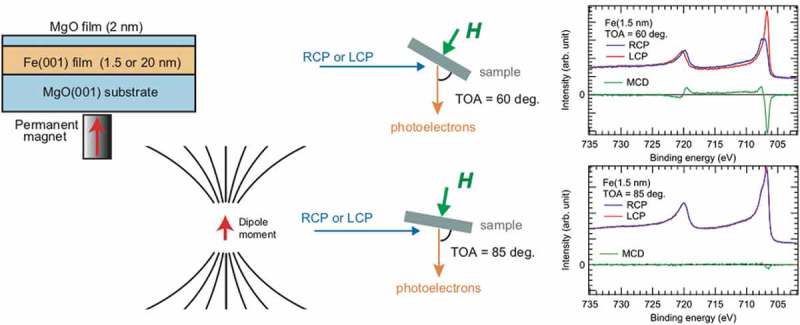

## 1. Introduction

Ferromagnetic metal (FM) thin films having a magnetization easy axis perpendicular to the film surfaces due to a strong magneto-crystalline anisotropy (MCA) have attracted much attention in spintronic applications, since thermally stable perpendicular magnetization sufficiently above room temperature (RT) is required to realize non-volatile ultrahigh-density magnetic storage devices. It is well known that *L*1_0_-ordered FePt(001) and CoPt(001) thin films exhibit the perpendicular magnetic anisotropy (PMA) along the *c*-axis due to the large uniaxial magnetic anisotropy (*K*_u_) of these materials [1–3]. Although the growth of the *L*1_0_-FePt and CoPt thin films has been established by several groups [1–3], the films contain precious Pt metal that is a disadvantage in cost for applications. For the development of spintronic device applications in practical ways, it is necessary that the devices are made from earth-abundant and environmentally friendly materials.

Ikeda et al. reported that a magnetic tunnel junction (MTJ) of such materials in the Ta/CoFeB/MgO/CoFeB/Ta structure with ultrathin CoFeB films (1.3 nm in thickness) showed the PMA at the CoFeB/MgO interface [4]. They demonstrated the spin-transfer torque (STT) magnetization switching by a low threshold current for the perpendicular CoFeB/MgO MTJ with the lateral size of 40 nm in diameter. Since the MTJ is one of the structural elements of magnetoresistive random access memory (MRAM) devices, the STT magnetization switching is important to realizing low-energy consumption MRAM devices. In addition, the perpendicular MTJs with small lateral size can contribute to realize ultrahigh-density MRAM devices.

Currently, the interface-induced PMA has been experimentally reported in several MgO/FM interfaces, including the dependence on the buffer layer materials for the FM thin films [4–8]. Okabayashi et al. reported the experimental results of magnetic circular dichroism (MCD) in X-ray absorption spectroscopy (XAS) [XMCD] to investigate the interface PMA for MgO/Co_2_FeAl and MgO/Fe, as judged from orbital magnetic moments [7,8]. From the angular dependence of the orbital moment in Fe, they claimed that the interface PMA is mainly induced by the orbital moment of Fe at the interfaces. Although XMCD is a powerful tool for detecting the element specific spin and orbital magnetic moments via the magneto-optical sum-rule analysis [9,10], it does not directly give the electronic structures of FMs near the interfaces. The electronic structures of FMs near the interfaces are important to understand the origin of the interface-induced PMA and have been studied by the first-principles calculations [11–14]. Thus, the direct observation of the electronic structure of FMs near the interfaces is a key to understanding the interface-induced PMA. We have thus performed hard X-ray photoelectron spectroscopy (HAXPES), which is a direct probe of the electronic structures of solids and can probe a buried interface due to a large probing depth [15–18], for MgO/Fe/MgO(001) structures. A direct observation of the electronic structure for a MgO/Fe interface with perpendicular magnetization will provide new information on the interface PMA, because the MgO/Fe interface is a prototypical example of the interface PMA and the materials used in the MgO/Fe interface are of low cost, earth abundant, and environmentally friendly, which are demands for sustainable device applications.

**Figure 1.**
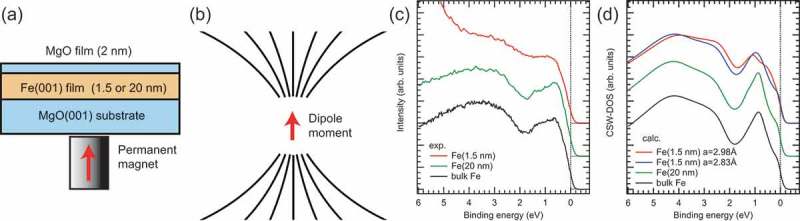
(a) Schematic illustration of the MgO (2 nm)/Fe (1.5 and 20 nm)/MgO(001) structure with a permanent magnet for magnetization. (b) Schematic diagram of magnetic-field-lines from the magnetic dipole moment. The magnetization is indicated by red arrows in (a) and (b). (c) Experimental valence band HAXPES spectra of the MgO (2 nm)/Fe (1.5 and 20 nm)/MgO(001) structures and bulk polycrystalline Fe. (d) Simulated valence band HAXPES spectra (CSW-DOSs) of the MgO (2 nm)/Fe (1.5 and 20 nm)/MgO(001) structures and bulk Fe. Upturn of the intensity at E_B_~4.5 eV in the experimental spectrum for the 1.5-nm-thick film is due to the substrate MgO-derived states, which are not included in the simulation.

The sample structures used in this work are shown in Figure 1(a). The top MgO (2 nm) film is used to form the MgO/Fe interface and acts as a protection film to prevent oxidation after the sample fabrication. For the MgO (2 nm)/Fe (1.5 nm)/MgO(001) structure, HAXPES probes both the top and bottom MgO/Fe interfaces, while it mainly probes the Fe film inside for the MgO (2 nm)/Fe (20 nm)/MgO structure, since the inelastic mean free path (IMFP) of photoelectrons with 6 keV in Fe (MgO) is 6.8 (9.2) nm [19]. That is, the ratio of the photoemission intensity from the Fe 1 monolayer (ML) contacting with the top MgO film (i.e. top MgO/Fe interface) to the total photoemission intensity from the Fe film is very weak (~2%) to detect in the HAXPES experiments by considering the photoemission intensity distribution in the Fe film as a function of the depth (*d*) from the interface; exp(-*d*/IMFP). Although the top MgO film acts as a protection film, we have to measure the HAXPES spectra of the samples as fresh as possible to obtain the reliable valence-band spectra relating to the MgO/Fe interface. The quality of the samples is quite important for correctly understanding the mechanism of PMA from the valence-band electronic states. Therefore, the HAXPES measurements were performed before analysing the magnetic properties of the samples. We have developed MCD in HAXPES (MCD-HAXPES) [20] under an applied magnetic field of 1 kOe to *in situ* clarify the electronic and magnetic states of the samples. MCD-HAXPES for the remanently magnetized samples is unfavourable for detecting the perpendicular magnetization, since the coercivity of MgO/FM interfaces is generally small [4–8] and the demagnetization would occur by stray fields during transportation in ultra-high vacuum (UHV) chambers.

**Figure 2.**
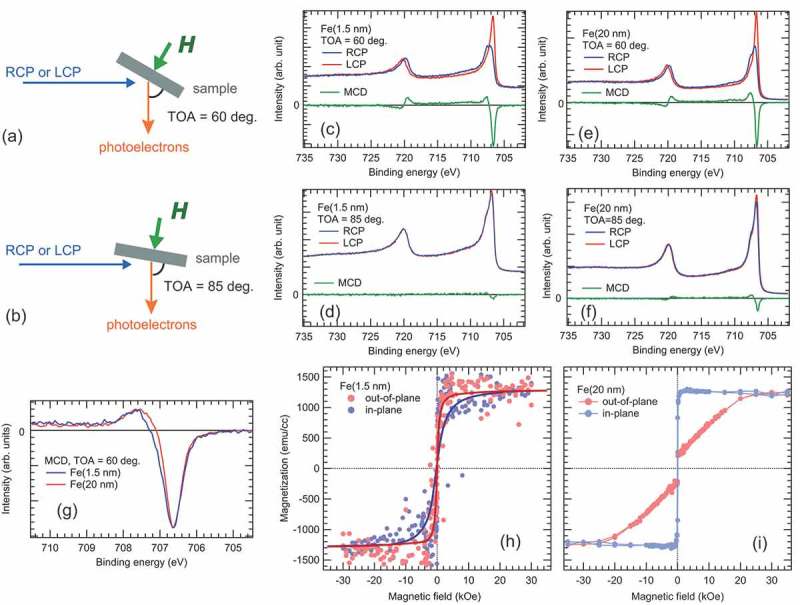
Schematic diagram of the experimental geometry of MCD-HAXPES measurements at TOA = 60° (a) and 85° (b). The magnetic field at the sample surface is 1 kOe (0.1 T) and is perpendicular to the sample surface in both cases. The Fe 2*p* core-level MCD-HAXPES spectra of the MgO (2 nm)/Fe (1.5 nm)/MgO(001) structure measured at TOA = 60 and 85° are shown in (c) and (d), respectively. The MCD-HAXPES spectra of the MgO (2 nm)/Fe (20 nm)/MgO structure measured at TOA = 60 and 85° are shown in (e) and (f), respectively. (g) Comparison of the MCD spectra for MgO (2 nm)/Fe (1.5 and 20 nm)/MgO structures measured at TOA = 60°. The hysteresis curves of MgO (2 nm)/Fe (1.5 and 20 nm)/MgO structure are shown in (h) and (i), respectively. Thick solid lines are to guide the eye. For the hysteresis curve measurements (h), the magnetic field step was set to 50, 200, and 1000 Oe in the range of 0–0.4, 0.4–5, and 5–30 kOe, respectively. For the in-plane hysteresis curve measurement (i), the field step was set to 200, 1000, and 5000 Oe in the range of 0–1, 1–10, and 10–50 kOe, respectively, while for the out-of-plane hysteresis curve measurement (i), the field step was set to 100, 500, 1000, and 5000 Oe in the range of 0–2, 2–10, 10–15 and 15–50 kOe, respectively.

However, to perform the PES measurements in a magnetic field, a serious problem should be solved; the photoelectron trajectory is easily affected by the presence of magnetic field, through the Lorentz force [***F***(***r***) = *q**v***(***r***) × ***B***(***r***)], where *q*, ***v, B***, and ***r*** are the charge of electron, velocity of electron, magnetic flux density, and the distance with respect to the centre of magnetic dipole, respectively. To eliminate the quite weak magnetic field (~0.5 Oe) from the Earth, PES experiments are generally performed in a magnetically shielded chamber. To overcome this problem, we have designed an experimental geometry for HAXPES under a magnetic field that is produced by a permanent magnet (see Figure 1(a)). Figure 1(b) shows the magnetic field lines radiating from the magnetic dipole. Trajectories of photoelectrons moving along magnetic field lines are less affected by the magnetic field owing to the fact that ***F***(***r***) is almost zero, that is, ***B***(***r***) is nearly parallel to ***v***(***r***). We have assessed this assumption experimentally, and that photoelectrons at around 6 keV emitted within ±30° with respect to the direction of the dipole moment appears to satisfy the condition of ***B***(***r***) nearly parallel to ***v***(***r***), which allows us to perform HAXPES under a magnetic field in the experimental geometries shown in Figure 2(a) and 2(b). In addition, the steep decrease of the magnetic field, which is proportional to ~1/*r*^3^, is also crucial for several keV photoelectrons to reduce the changes of photoelectron trajectories.

## 2. Experiment

1.5 and 20-nm-thick Fe thin films were fabricated by molecular beam epitaxy using an electron beam evaporator on MgO(001) substrates in an UHV chamber with the base pressure of 1.0 × 10^−7^ Pa at RT. After the deposition, samples were annealed at 300°C in UHV to obtain a good crystallinity. An epitaxial growth was clarified from the reflection high-energy electron diffraction patterns after the annealing process. Finally, a 2-nm-thick MgO film was grown on the Fe films at RT to form the top MgO/Fe interface and to avoid the surface oxidization of the Fe films. We note that all the thicknesses are given in nominal values.

The HAXPES measurements at RT were performed at the undulator beamline BL15XU [18,21] of SPring-8, Japan. The excitation photon energy was fixed at 5.95 keV. The photoelectrons were analysed and detected by a high-resolution hemispherical analyser (VG Scientia R4000). The angle between the objective lens of the analyser and photon propagation was fixed at 90°. In order to apply a magnetic field, a Nd-Fe-B based permanent magnet (2.5 mm in diameter with a 2-mm-thickness) was placed under a sample. The magnetic field was applied perpendicular to the sample surface and the measured magnetic field at the sample surface was 1.0 kOe. Total energy resolution was set to 240 meV, which was confirmed by the Fermi cut-off of an evaporated Au thin film. For the Fe 2*p* core-level MCD-HAXPES measurements, horizontal linear polarized (H-pol.) X-rays were converted to left-or-right-handed circularly polarized (LCP or RCP) X-rays by using a diamond phase retarder. The degree of circular polarization was estimated to be 0.95. For the valence-band measurements, H-pol. X-rays from the undulator were used with the degree of linear polarization of ~1.00. A clean surface of polycrystalline bulk Fe as a reference sample was obtained with a diamond file in an UHV chamber, while no surface treatment was done for the MgO (2 nm)/Fe (1.5 and 20 nm)/MgO structures. The binding energy (*E_B_*) was referred to the Fermi-level (*E_F_*) of an evaporated Au film. We carefully checked for charging effects for the 1.5-nm-thick Fe film, since the photoelectron emission from the insulating MgO substrate was dominant in this case, and such effects were found to be negligible.

The magnetic hysteresis curves for the MgO (2 nm)/Fe (1.5 and 20 nm)/MgO(001) structure were measured by a superconducting quantum interference device (SQUID) magnetometer (Quantum Design, MPMS2) at RT after the HAXPES measurements. The applied magnetic field direction is parallel (in-plane) or perpendicular (out-of-plane) to the film surface.

## 3. Results and discussion

Figure 2 shows the Fe 2*p* core-level HAXPES spectra of the MgO (2 nm)/Fe (1.5 and 20 nm)/MgO(001) structures under a magnetic field of 1 kOe (see Figure 2(a) and 2(b) for experimental geometries) using RCP and LCP X-rays. MCD is the intensity difference between the RCP and LCP spectra, shown as green curves. Since the MCD signal is proportional to the magnetization projected to the direction of X-rays, the geometry shown in Figure 2(b) is sensitive to the in-plane magnetization, while that shown in Figure 2(a) is sensitive to both the in-plane and perpendicular magnetizations. For the 1.5-nm-thick film, the strong and negligible weak MCD signals for take-off-angles (TOAs) of 60° and 85° were seen in Figure 2(c) and 2(d), respectively. This result is clear evidence of the perpendicular magnetization. For the 20-nm-thick film, MCD signals were clearly seen in the spectra for both TOAs (see Figure 2(e) and 2(f)). Figure 2(g) compares the MCD spectra of the Fe films for TOA of 60°. The energy splitting between the peak and valley is slightly larger in the 1.5-nm-thick film (1.0 eV) than the 20-nm-thick film (0.95 eV). This result indicates a slight enhancement of the Fe 3*d* magnetic moment in the 1.5-nm-thick film, since the energy splitting is caused by the spin-exchange interaction between the Fe 2*p* core-hole and 3*d* electrons in the photoemission final states [22]. For both films, the Fe 2*p* spectra do not show the Fe^2+^ and Fe^3+^ components [20,23], suggesting that the high-quality MgO/Fe interfaces are formed without extrinsic oxidation in the Fe films. It has been reported that an additional AlO*_x_* capping film (~1 nm) was deposited to protect a MgO film against moisture in air [24,25]. Although we did not use an additional AlO*_x_* capping film, our experimental results indicate that the MgO/Fe interfaces are not affected by moisture in air during transportation.

The ratio of the MCD signal to the Fe 2*p*_3/2_ core-level peak height for TOA = 60° at *E_B_* around 706.5 eV for the 20-nm-thick Fe film (35% in the intensity asymmetry) is smaller than that for the 1.5-nm-thick Fe film (43%), suggesting unfavourable perpendicular magnetization in the 20-nm-thick film due to the shape anisotropy. The weak MCD signal in Figure 2(f) could originate from magnetic moments randomly lying in the fourfold in-plane easy magnetization axes along [100] and [010], forming multi-domain structures. Figure 2(h) and 2(i) show the *ex situ* measured hysteresis curves after the HAXPES measurements. The easy axis for the 1.5-nm-thick film was out-of-plane (perpendicular) with *K*_u_ = 11.95 × 10^6^ erg/cm^3^, which exceeded the shape anisotropy of 9.97 × 10^6^ erg/cm^3^ estimated from the magnetic moment of the Fe film, and the magnetization steeply saturated by a low magnetic field. For the 20-nm-thick film, the easy axis was in-plane with *K*_u_ = 0.07 × 10^6^ erg/cm^3^, while a small out-of-plane magnetization component at a low magnetic field was seen in the hysteresis curve. This indicates the formation of multi-domain structures and non-collinear magnetization in the 20-nm-thick Fe film, as reported by Kawauchi et al. [26].

To clarify the electronic structures of the MgO (2 nm)/Fe (1.5 nm)/MgO(001) structure, which exhibited the perpendicular magnetization, we compared the valence-band HAXPES spectra of the 1.5- and 20-nm-thick Fe films for TOA of 88° measured with H-pol. X-rays. As seen in Figure 1(c), the spectral shape for the 20-nm-thick Fe film is almost identical to that for bulk Fe. The peak structure located at *E_B_* range from 0 to 2 eV and broad peak located at *E_B_* around 3.5 eV are mainly due to the Fe 3*d* and 4*s* states, respectively [22]. For the 20-nm-thick Fe film, HAXPES mainly probes the Fe film inside due to the large IMFP as mentioned above. Thus, the similarity of the valence-band spectra indicates that the Fe lattice constant of the thick Fe film almost relaxed to that of bulk Fe. The 3*d* orbitals are expected to split into degenerate *e*_g_ (3*z*^2^-*r*^2^ and *x*^2^-*y*^2^) and *t*_2g_ (*xy, yz*, and *zx*) orbitals inside of the Fe film as well as in bulk Fe.

For the 1.5-nm-thick Fe film, we found that the Fe 3*d* derived states are broadened and expand to the higher *E_B_* side, in comparison with those of the 20-nm-thick film and bulk Fe. This result arises from the fact that the interface-derived Fe electronic states are emphasized in the spectrum for the 1.5-nm-thick Fe film because the top MgO film thickness of 2 nm and the Fe film thickness of 1.5 nm (~10 ML) are sufficiently thin to permit probing both the top and bottom MgO/Fe interfaces by HAXPES. It is considered that the lifting of degeneracy in the Fe 3*d* states due to the MgO/Fe interfaces is detected in the valence-band spectrum. According to previous work [12,13], the hybridization between the Fe 3*d*(3*z*^2^-*r*^2^) and O 2*p*(*z*) orbitals at the MgO/Fe interface causes the lifting of degeneracy in the Fe 3*d* states, contributing to the interface PMA. In addition, for the thin Fe film, the in-plane lattice constant of Fe would be affected by the tensile strain from the MgO substrate due to the lattice mismatch (5%) of bulk Fe and MgO. Therefore, the valence-band spectrum reflects the lifting of degeneracy in the Fe 3*d* states caused by the combination of Fe 3*d*-O 2*p* hybridization at the interfaces and the tensile strain in the Fe film. Thus, the direct observation of the valence-band spectrum for the MgO (2 nm)/Fe (1.5 nm)/MgO(001) structure helps us understand the MgO/Fe interface PMA.

To understand our results better theoretically, we have carried out density functional theory (DFT) calculations to obtain the density of states (DOS) for MgO/Fe/MgO(001) structures and to clarify the relationship between the electronic structures and PMA. We used the projector augmented wave method as implemented in the Vienna *ab initio* simulation package [27]. The generalized gradient approximation as parameterized by Perdew, Burke, and Ernzerhof is used for the exchange correlation potential [28]. An energy cutoff of 500 eV is used for the plane-wave expansion. We constructed two MgO (7 ML)/Fe (15 ML) multilayer structures to study the electronic structure of MgO/Fe interface. The in-plane lattice constant of the Fe layers in the multilayer structure is fixed to i) *a* = 2.98 Å (match to the bulk MgO lattice constant) and ii) *a* = 2.83 Å (the bulk bcc-Fe lattice constant). In both the multilayer structures, the atomic position and cell structure along *c*-axis are optimized until the atomic force and stress are less than 0.03 eV/Å and 0.01 MPa, respectively. The 12 × 12 × 1 and 16 × 16 × 2 Monkhorst-Pack grids for k-point are used for the calculation of geometry optimization and DOS, respectively. We also calculated the DOS for bulk bcc Fe in comparison with the DOSs of Fe in the multilayer structure by using 16 × 16 × 16 k-point mesh. The magnetic anisotropy energy (MAE) of the MgO/Fe multilayer is evaluated using the magnetic force theorem [29]. A 24 × 24 × 3 k-point mesh is used for the estimation of the MAE.

**Figure 3.**
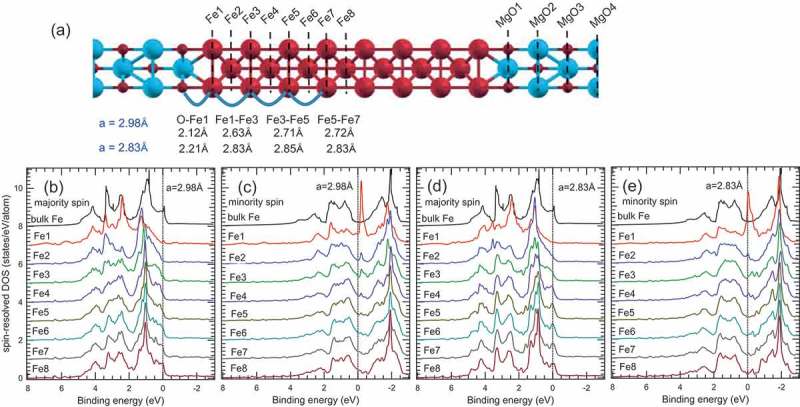
(a) Schematic diagram of the MgO (7 ML)/Fe (15 ML) multilayer structure for the DFT calculations. The spacing of Fe-O and Fe–Fe for *a* = 2.98 and 2.83 Å is also shown in the figure. The calculated Fe layer-resolved DOSs of the MgO (7 ML)/Fe (15 ML) multilayer structure with *a* = 2.98 Å for (b) the majority and (c) minority spin states. The calculated Fe layer-resolved DOSs of the superlattice structure with *a* = 2.83 Å for (d) the majority and (e) minority spin states. The Fe layer-resolved DOSs are broadened by a Gaussian function with FWHM of 0.1 eV for visibility. The calculated spin-resolved DOSs for bulk Fe are shown for comparison. The dotted lines indicate the Fermi-level.

Figure 3(a) shows the calculated MgO (7 ML)/Fe (15 ML) multilayer structure for *a* = 2.98 and 2.83 Å with consideration of the relaxation of the out-of-plane lattice parameters. The Fe layer- and spin-resolved DOSs for the multilayers with *a* = 2.98 and 2.83 Å and DOSs of bulk Fe are also shown in Figure 3(b–e). The spin-resolved DOSs of the Fe1 layer are strongly modified from those of bulk Fe for both *a* = 2.98 and 2.83 Å. This result is mainly explained by the lifting of degeneracy in the Fe 3*d* states owing to the hybridization between the Fe 3*d*(3*z*^2^-*r*^2^) and O 2*p*(*z*) orbitals at the MgO/Fe interface [12,13]. Although the Fe 3*d*-O 2*p* hybridization occurs in the Fe1 layer, this result does not suggest an oxidation of the Fe1 layer. In fact, the metallic DOS still remains in the Fe1 layer. The modification of the spin-resolved DOSs of the Fe2 layer is also found for both *a* = 2.98 and 2.83 Å. The difference between the DOSs of the Fe layer and bulk Fe becomes immediately smaller with increasing the Fe layer number. In particular, the spin-resolved DOSs of the Fe5-8 layers for *a* = 2.83 Å are almost identical to those of bulk Fe.

Table 1 summarizes the calculated Fe layer-resolved MAE. Here, the positive (negative) MAE contributes to perpendicular (in-plane) magnetization. The MAE is largest in the Fe1 layer for both *a* = 2.98 and 2.83 Å and steeply decreases with increasing the Fe layer number. The PMA of the Fe1 and Fe2 layer for *a* = 2.98 Å is significantly larger than that for *a* = 2.83 Å as a consequence of the strong tetragonal distortion seen in Figure 3(a). Thus, we can conclude that the tetragonal distortion and the electronic states near the MgO/Fe interface play important roles in the interface PMA.

**Table 1.** Magnetic anisotropy energy of Fe (μeV/atom).

*a* (Å)	Fe1	Fe2	Fe3	Fe4	Fe5	Fe6	Fe7	Fe8
2.98	719	169	−18.1	66.9	−24.2	25.1	−7.2	−23.1
2.83	419	10.6	−9.5	25.9	1.9	−7.2	1.8	−0.1

Table 2 shows the calculated Fe layer-resolved spin and orbital magnetic moments. The spin and orbital magnetic moments are largest in the Fe1 layer for *a* = 2.98 Å, and decrease with increasing the Fe layer number as well as those for *a* = 2.83 Å. For *a* = 2.83 Å, the magnetic moment of the Fe layer approaches that of bulk Fe as the layer number increases, which originates from that the DOSs of the Fe5-8 layers are almost identical to those of bulk Fe. As a result of the tetragonal distortion together with the Fe 3*d*-O 2*p* hybridization, the lifting of degeneracy in the Fe 3*d* states occurs in the Fe layers, leading the larger magnetic moment in the Fe layers for *a* = 2.98 Å than *a* = 2.83 Å. However, the magnetic moment of Fe in the MgO (2 nm)/Fe (1.5 nm)/MgO structure is enhanced by a few per cent, if any, in comparison with that in the MgO (2 nm)/Fe (20 nm)/MgO structure (bulk-like Fe), when we evaluate the Fe magnetic moment from the energy splitting between the negative and positive MCD signals shown in Figure 2(g). The XMCD study on the MgO/Fe interface also did not show the strong enhancement of the Fe magnetic moment that has been suggested in the literature [8]. Although it is difficult to recognize the strong enhancement of the magnetic moment of Fe from the experiments, we can again conclude that the tetragonal distortion and the electronic states at the MgO/Fe interface play important roles in the interface PMA.

**Table 2.** Magnetic moment of Fe (μ_B_/atom).

*a* (Å)		Fe1	Fe2	Fe3	Fe4	Fe5	Fe6	Fe7	Fe8
2.98	Spin	2.756	2.513	2.498	2.463	2.436	2.417	2.415	2.400
	Orbital	0.075	0.053	0.051	0.050	0.048	0.049	0.049	0.048
2.83	Spin	2.785	2.356	2.383	2.254	2.181	2.189	2.161	2.187
	Orbital	0.109	0.056	0.050	0.048	0.045	0.046	0.045	0.046

**Figure 4.**
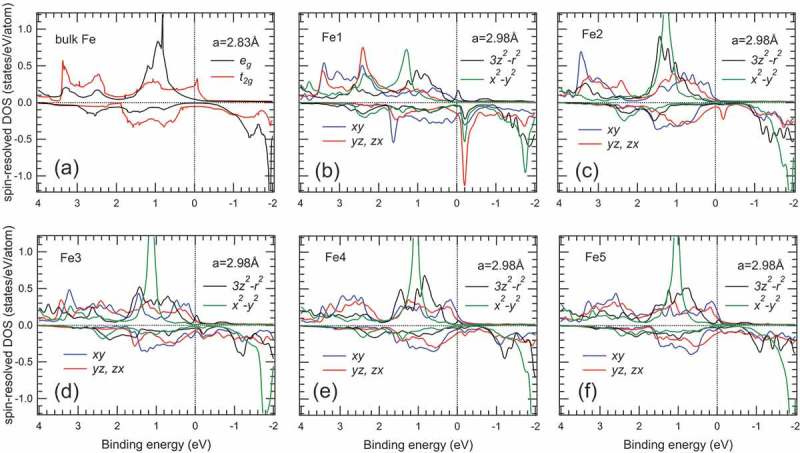
Calculated spin-resolved 3*d* DOSs for (a) bulk Fe and (b)-(f) Fe1-Fe5 layers in the multilayer structure with *a* = 2.98 Å, respectively. The 3*d* DOSs for the Fe1-Fe5 layers are broadened by a Gaussian function with FWHM of 0.1 eV for visibility. The dotted lines indicate the Fermi-level.

Figure 4 shows the spin-resolved 3*d* DOSs of the Fe1-Fe5 layers for *a* = 2.98 Å and bulk Fe. The 3*d* orbitals degeneracy is split into the *e*_g_ and *t*_2g_ states in bulk Fe as mentioned above. In contrast, the lifting of the degeneracy in the 3*d* orbitals occurs in all the Fe layers for the MgO/Fe multilayer structure, while the *yz* and *zx* orbitals are still degenerate, originating from the tetragonal distortion of the Fe lattice. The Fe 3*d* DOSs of the Fe1 layer are strongly modified from those of bulk Fe due to the Fe 3*d*(3*z*^2^-*r*^2^)-O 2*p*(*z*) hybridization and tetragonal distortion as mentioned above. The Fe 3*d* DOSs of the Fe2 layer are slightly affected by the Fe1 layer through the *yz-yz* and *zx-zx* orbital hybridizations between the Fe1 and Fe2 layers.

To understand the valence-band spectrum of the MgO (2 nm)/Fe (1.5 nm)/MgO(001) structure, which showed the perpendicular magnetization, we have simulated the HAXPES spectrum by using the Fe 3*d*, 4*s*, and 4*p* partial DOSs obtained by the DFT calculations and the photoionization cross-sections for the 3*d*, 4*s*, and 4*p* orbitals. For comparison, we have also simulated the HAXPES spectra of the MgO (2 nm)/Fe (20 nm)/MgO structure and bulk Fe. The simulated HAXPES spectra correspond to the cross-section weighted DOSs (CSW-DOSs). The per electron cross-section ratio of the 4*s* (4*p*) orbital to the 3*d* orbital is set to 50 (1.3), where the 4*s* cross-section is twice of the calculated cross-section [30–32] to better reproduce the experimental valence-band spectra. In the simulations, the CSW-DOSs are broadened by a Lorentzian function (FWHM varying ~0.2 × *E_B_* (eV)) [33–35]. Then, the CSW-DOSs are multiplied by the Fermi-Dirac function for RT and are finally broadened by a Gaussian function (FWHM of 0.24 eV).

Figure 1(d) shows the simulated valence-band HAXPES spectra for 1.5- and 20-nm-thick Fe films and bulk Fe. For the 1.5-nm-thick Fe films (~10 ML), the DOSs of the Fe1-Fe5 layers for *a* = 2.98 Å are considered in the simulation for taking the two MgO/Fe interfaces into account, since HAXPES probes both the top and bottom MgO/Fe interfaces. The DOS of the Fe8 layer for *a* = 2.83 Å is used in the simulation for the 20-nm-thick Fe film, because HAXPES mainly probes the Fe film inside as mentioned above. The CSW-DOSs well reproduce the experimental valence-band spectra (see Figure 1(c) and 1(d)). We note that the CSW-DOS calculated from the Fe1-Fe5 layers for *a* = 2.98 Å (red curve) better reproduces the experimental spectrum than that for *a* = 2.83 Å (blue curve), suggesting that the effect of the tetragonal distortion cannot be negligible in the electronic structure of the 1.5-nm-thick Fe film. Thus, we see that the broad valence-band spectrum for the 1.5-nm-thick Fe film observed in HAXPES is strongly affected by the lifting of degeneracy in the Fe 3*d* states due to the Fe 3*d*-O 2*p* hybridization at the interface and tetragonal distortion of the Fe film.

In this paper, we discussed the electronic and magnetic states of the Fe film near the MgO/Fe interface by comparing the valence-band and Fe 2*p* core-level spectra for the MgO (2 nm)/Fe (1.5 and 20 nm)/MgO(001) structures. In contrast, Yang et al. [24] reported soft X-ray standing-wave (SW) PES for the AlO*_x_* (~1.2 nm)/MgO (~1 nm)/wedged-Fe (0–20 nm) structure on the [MoSi_2_/Si]_80_ multilayer with 4.98 nm periodicity. Since the SW period is same as the period of the multilayer and typical resolution in depth is ~1/10 of the period, they were able to derive the depth-resolved sample structure and magnetization profile for the in-plane magnetization component near the MgO/Fe interface from SW-PES experiments. Although the sample fabrication method, sample structure, and photon energy in Ref [24] are different from this work, it is expected that the SW technique with HAXPES yields more precise depth distribution of electronic and magnetic states with a few Å resolution in depth near the MgO/Fe interface, as a future direction.

## 4. Summary

We have developed HAXPES in an applied magnetic field of 1 kOe to clarify the electronic and magnetic states of the spintronic structures and applied the technique in this work to the MgO (2 nm)/Fe (1.5 and 20 nm)/MgO(001) structures. For the MgO (2 nm)/Fe (1.5 nm)/MgO(001) structure, perpendicular magnetization of the Fe film without extrinsic oxidation was confirmed by the Fe 2*p* core-level MCD-HAXPES measurements under a magnetic field, and the easy magnetization axis was perpendicular to the film plane, which was confirmed by the *ex situ* in-plane and out-of-plane magnetic hysteresis curve measurements. For the MgO (2 nm)/Fe (20 nm)/MgO(001) structure, the Fe 2*p* core-level MCD-HAXPES and magnetic hysteresis curve measurements suggested non-colinear magnetization with multi-domain structures and in-plane easy magnetization axis. By confirming the perpendicular magnetization of the MgO (2 nm)/Fe (1.5 nm)/MgO(001) structure without extrinsic oxidation of the Fe film from the Fe 2*p* core-level MCD-HAXPES under a magnetic field, we directly obtained reliable valence-band HAXPES spectrum of the Fe film with the perpendicular magnetization for the first time. Combining experiment with DFT indicates that the valence-band spectrum clearly showed the lifting of degeneracy in the Fe 3*d* states due to the tetragonal distortion of the Fe film by the MgO substrate and the Fe 3*d*-O 2*p* hybridization at the MgO/Fe interfaces, although the top and bottom interfacial Fe layers (2 ML) are ~20% in the 1.5-nm-thick Fe film (~10 ML). The simulated HAXPES spectrum using the DOSs obtained by DFT for MgO (7 ML)/Fe (15 ML) multilayer structure with *a* = 2.98 Å well reproduce the experimental valence-band spectrum for the 1.5-nm-thick Fe film. Thus, we can conclude that the tetragonal distortion in the Fe film and the Fe 3*d*-O 2*p* hybridization at the MgO/Fe interface play important roles in the interface PMA.

The electronic structure measurements by HAXPES under a magnetic field can open the studies on the perpendicularly magnetized FMs and materials having magnetic field-induced phase transitions. Further development of HAXPES experiments under a higher magnetic field (>10 kOe) expands the materials science from the viewpoint of the electronic structures of solids. Since HAXPES measurements under an electric field have already been done by one of the authors [18,36,37], HAXPES under a coexistence of electric and magnetic fields will be developed in near future and can be a powerful tool for studying the materials for spintronic applications [11,13,38,39] to understand the phenomena driven by electric and magnetic fields from the electronic structures.
